# Three new species of *Rhaphium* from China, with an updated key to Chinese *Rhaphium* (Diptera, Dolichopodidae, Rhaphiinae)

**DOI:** 10.3897/zookeys.840.31602

**Published:** 2019-04-17

**Authors:** Qilemoge, Mengqing Wang, Ding Yang

**Affiliations:** 1 Department of Entomology, College of Plant Protection, China Agricultural University, Beijing 100193, China China Agricultural University Beijing China; 2 Institute of Plant Protection, Chinese Academy of Agricultural Sciences, Beijing 100193, China Institute of Plant Protection, Chinese Academy of Agricultural Sciences Beijing China

**Keywords:** China, new species, *
Rhaphium
*

## Abstract

At present, there are 26 species in the genus *Rhaphium* Meigen known from China. In this paper, three species from China are described as new to science: *Rhaphiumgangchanum***sp. n.**, *Rhaphiumshaliuhense***sp. n.**, and *Rhaphiumtianshuiense***sp. n.** A key to the Chinese species of *Rhaphium* is provided.

## Introduction

The genus *Rhaphium* Meigen belongs to the subfamily Rhaphiinae (Dolichopodidae) with 199 known species worldwide (Yang et al. 2006, 2011; Grichanov 2017). Twenty-six species have been recorded from China, with nine species only from Oriental China, 14 species only from Palaearctic China, two species from Oriental and Palaearctic China, and *R.dilatatum* Wiedemann, 1830 with an unclear Chinese distribution.

The specimens upon which this study is based were collected in Beijing as well as the Hebei, Qinghai, and Gansu regions of China. Gansu and Qinghai Provinces are located in northwestern China. Gansu Province has a temperate monsoon climate and lies between the Tibetan Plateau and the Loess Plateau. Qinghai Province has a plateau continental climate and is located in the northeastern part of the Tibetan Plateau. Beijing, the capital of China, is located in the north of the country and has a subtemperate climate. Hebei Province is also in the north, and its the climate is similar to that of Beijing. In the present paper, we describe three new species of the fauna of China and provide a key to all species of Chinese *Rhaphium*, except for *R.dilatatum* Wiedemann, 1830 and *R.relates* (Becker, 1922) because they are poorly described and the whereabouts of the holotypes are unknown.

## Material and methods

The specimens in this study were collected by sweep nets and subsequently stored into 95% ethanol. All specimens are deposited in the Entomological Museum of China Agricultural University (CAU), Beijing. Morphological terminology for adult structures mainly follows Cumming and Wood (2009). The following abbreviations are used: **acr** = acrostichal bristle(s), **ad** = anterodorsal bristle(s), **av** = anteroventral bristle(s), **cer** = cercus, **CuAx ratio** = length of m-cu / length of distal portion of CuA, **dc** = dorsocentral bristle(s), **npl** = notopleural bristle(s), **oc** = ocellar bristle(s), **pa** = postalar bristle(s), **pd** = posterodorsal bristle(s), **pvt** = postvertical bristle(s), **sa** = supraalar bristle(s), **sc** = scutellar bristle(s), **sur** = surstylus, **vt** = vertical bristle(s).

## Taxonomy

### Rhaphium

Taxon classificationAnimaliaDipteraDolichopodidae

Meigen, 1803

Rhaphium Meigen 1803: 272. Type species: Rhaphiummacrocerum Meigen, 1824.

#### Diagnosis.

Body small to large (1.5–5.7 mm); vertex flat; oc nearly as long as vt; face obviously narrower than frons; clypeus not obviously separate from face; antenna black, first flagellomere mostly prolonged, 2–10 times longer than wide, arista apical; propleuron with dense pale white hairs, without distinct bristle; hind coxa with or without 1 outer bristle at middle; vein M straight and not bifurcated, R_4+5_ parallel or slightly convergent with M in wing apex, CuAx ratio less than 1; abdominal segments 1–3 usually with long pale hairs, abdominal segment 6 visible and pubescent; male genitalia connected tightly with pregenital segment, cap-like, cercus varied, often long and narrowed towards tip, sometimes bifurcate, with hairs and bristles at middle (Yang et al. 2011).

### Key to species (males) of *Rhaphium* from China

**Table:** 

1	First flagellomere at least 4.0 times longer than wide (Fig. 6)	**2**
–	First flagellomere at most 2.5 times longer than wide (Fig. 4	**13**
2	Four dc	**3**
–	Five to six dc	**4**
3	First flagellomere about 4.3 times longer than wide (Yang et al. 2011: fig. 799); 2 or 3 pairs of acr; hairs and bristles on coxae black	***R.apicinigrum* Yang & Saigusa, 1999**
–	First flagellomere about 8.2 times longer than wide; acr absent (Yang et al. 2011: fig. 812); hairs and bristles on coxae pale yellow	***R.sichuanense* Yang & Saigusa, 1999**
4	Arista inflated apically (Yang et al. 2011: fig. 809)	***R.parentianum* Negrobov, 1979**
–	Arista simple, not inflated apically	**5**
5	Cercus bifoliate (Fig. 7)	**6**
–	Cercus not bifoliate	**8**
6	First flagellomere at most 7.0 times longer than wide (Fig. 6)	**7**
–	First flagellomere at least 9.0 times longer than wide	***R.bilobum* Tang, Wang & Yang, 2016**
7	All coxae black (Fig. 3)	***R.shaliuhense* sp. n.**
–	All coxae yellow	***R.daqinggouense* Tang, Wang & Yang, 2016**
8	First flagellomere at least 8.0 times longer than wide	**9**
–	First flagellomere at most 6.0 times longer than wide	**10**
9	Eight uniseriate acr; cercus nearly triangular, short, not bifurcated	***R.neimengense* Tang, Wang & Yang, 2016**
–	Five to eight irregularly paired acr; cercus deeply bifurcated into 2 long lobes (Yang et al. 2011: fig. 816)	***R.zhongdianum* Yang et Saigusa, 2001a**
10	Palpus yellow; acr absent; surstylus bifurcated apically (Yang et al. 2011: fig. 802)	***R.furcatum* Yang & Saigusa, 2000**
–	Palpus dark; acr present; surstylus not bifurcated apically	**11**
11	Five dc; surstylus long and thin, without apical incision	***R.palliaristatum* Yang & Saigusa, 2001b**
–	Six dc; surstylus short and wide, with apical incision	**12**
12	All coxae yellow; hind tibia yellow; surstylus with long thick hairs apically; cercus long band-like (Yang et al. 2011: fig. 815)	***R.xinjiangense* Yang, 1998a**
–	Only fore coxa yellow, mid and hind coxae black; hind tibia black; surstylus only with sparse short hairs; cercus long triangular (Yang et al. 2011: fig. 810)	***R.qinghaiense* Yang, 1998b**
13	Fore tarsus modified	**16**
–	Fore tarsus simple	**19**
14	Fore tarsomere 1 simple, fore tarsomere 5 with 2 Y-shaped apical bristles and 2 long strong bristles	***R.dorsiseta* Tang, Wang & Yang, 2016**
–	Fore tarsomere 1 modified, other various	**15**
15	Fore tarsomere 1 depressed dorsally but strongly raised ventrally	***R.lumbricus* Wei, 2006**
–	Fore tarsomere 1 inflated apically	**16**
16	Arista at least 1.4 as long as first flagellomere	**17**
–	Arista at most 0.8 as long as first flagellomere (Yang et al. 2011: fig. 813)	***R.sinense* Negrobov, 1979**
17	Fore tarsomere 2 inflated, mid tarsomeres 4 and 5 inflated (Yang et al. 2011: fig. 800)	***R.baihuashanum* Yang, 1998a**
–	Fore and mid tarsi simple, not inflated	**18**
18	Middle and lower postocular bristles yellow; 8 dc; mid femur yellow; cercus not bifoliate	***R.heilongjiangense* Wang, Yang & Masunaga, 2005**
–	All postocular bristles black; 5 dc; mid femur black; cercus bifoliate	***R.gangchanum* sp.n.**
19	Fore femur with row of strong ventral bristles or long ventral hairs	**20**
–	Fore femur without distinct ventral bristle or hairs	**21**
20	First flagellomere about 2.1 times longer than wide; arista about 1.9 times longer than first flagellomere; fore femur with row of long pale yellow ventral bristles as long as width of fore femur; cercus wide but narrow at base and widened onwards, with distinct marginal denticles (Yang et al. 2011: fig. 811)	***R.riparium* (Meigen, 1824)**
–	First flagellomere about 1.5 times longer than wide; arista about 2.8 times longer than first flagellomere; fore femur with 2 rows of long pale yellow bristles longer than width of fore femur; cercus very long, wide at basal half	***R.apophysatum* Tang, Wang & Yang, 2016**
21	Fore tarsus modified, tarsomere 1 with row of strong ventral bristles on basal half, tarsomere 2 inflated apically (Yang et al. 2011: fig. 807c)	**22**
–	Fore tarsus simple, tarsomere 1 without distinct ventral bristles, tarsomere 2 simple	**23**
22	Fore and mid femora yellow apically, fore and mid tibia yellow; fore coxa with black bristles and hairs	***R.micans* (Meigen, 1824)**
–	Fore femur, mid and hind tarsi dark; fore coxa with light yellow bristles and hairs	***R.dispar* Coquillett, 1898**
23	All coxae dark, fore and mid femora yellow apically	**24**
–	Basal half of fore coxa and apical 1/3 of hind femur dark	**25**
24	Hind tibia with 3 ventral bristles; mid tarsomere 1 1.1 times as long as hind tarsomere 1	***R.wuduanum* Wang, Yang & Masunaga, 2005**
–	Hind tibia without distinct ventral bristles; mid tarsomere 1 1.4 times as long as hind tarsomere 1	***R.gansuanum* Yang, 1998a**
25	Mid coxa with 1 strong outer bristle, and bunch of ventral bristles; mid tibia with 1 av	**26**
–	Mid coxa only with only 1 strong outer bristle at middle, without bunch of ventral bristles; mid tibia without ventral bristles	***R.bisectum* Tang, Wang & Yang, 2016**
26	Squama with yellow hairs; cercus not bifoliate; surstylus short and thick	***R.mediocre* (Becker, 1922)**
–	Squama with black hairs; cercus bifoliate; surstylus basally thick, apically sharp, with one protuberance (Fig. 9)	***R.tianshuiense* sp.n.**

### Rhaphium gangchanum sp. n.

Taxon classificationAnimaliaDipteraDolichopodidae

http://zoobank.org/F056D53D-892F-4AC0-BB88-EE81DA991605

Figures 1 , 4 , 5

#### Diagnosis.

First flagellomere about 2.2 times longer than wide; arista with basal segment 0.1 times as long as apical segment. All postocular bristles black. All coxae and femora black; fore tibia mainly yellow, black dorsally, mid tibia yellow, hind tibia mainly black, yellow at middle dorsally; fore tarsomere 1 inflated apically. Fore and mid coxae with black bristles, mid coxa apically with a bunch of black bristles, hind coxa with one black outer bristle. CuAx ratio 0.5. Surstylus short and thick, nearly square, with one apical protuberance. Cercus bifoliate, outer lobe and inner lobe strip-like, inner lobe 1/4 as long as outer lobe, apically with strong bristles.

#### Description.

**Figures 1–3.**
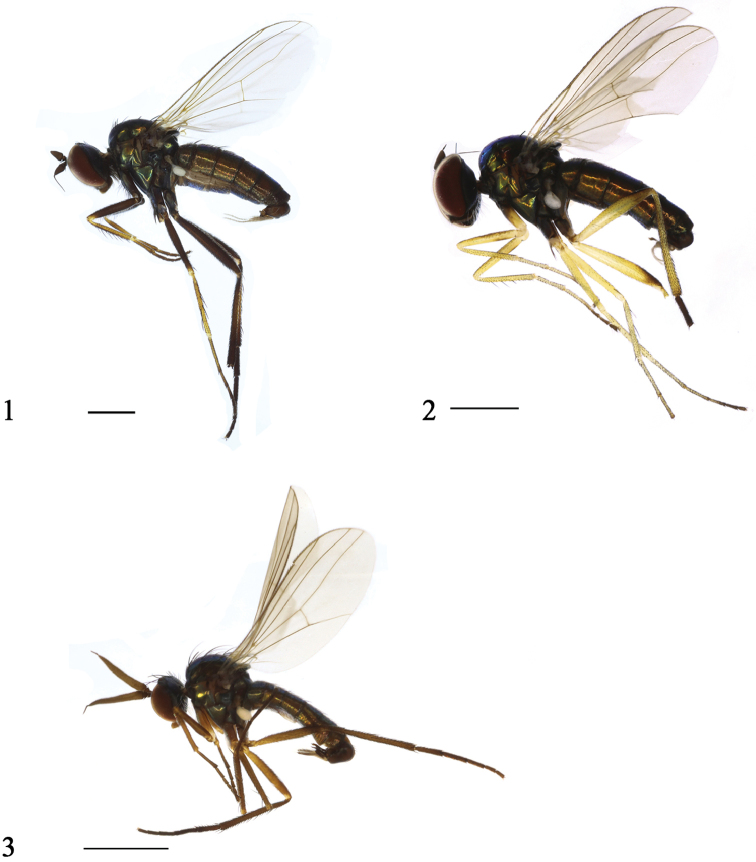
Habitus, lateral view. **1***Rhaphiumgangchanum* sp. n. male **2***Rhaphiumtianshuiense* sp. n., male **3***Rhaphiumshaliuhense* sp. n., male. Scale bars: 1 mm.

Male (Fig. 1). Body length 4.8 mm. Wing length 4.2 mm.

**Figures 4–7.**
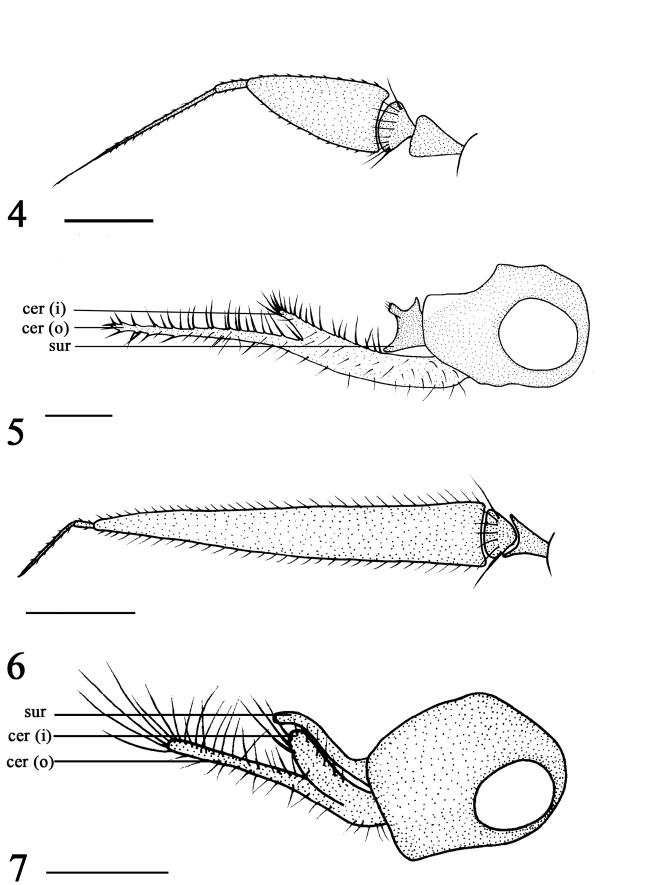
*Rhaphiumgangchanum* sp. n., male **4** antenna, lateral view **5** genitalia, lateral view. *Rhaphiumshaliuhense* sp. n., male **6** antenna, lateral view **7** genitalia, lateral view. Abbreviations: sur = surstylus, cer (o) = outer lobe of cercus, cer (i) = inner lobe of cercus. Scale bars: 0.2 mm.

Head metallic green with pale gray pruinescence. Frons with white pruinescence. Face black with pale pruinescence, not as wide as first flagellomere (length). All postocular bristles black. Two oc, two vt, two pvt. Antenna (Fig. 4) black; first flagellomere elongated, about 2.2 times longer than wide, apically sharp; arista black, apical position, basal segment 0.1 times as long as apical segment. Proboscis black with black hairs, palpus black with black apical bristle and hairs.

Thorax metallic green with pale gray pruinescence. Mesonotum without dark spot. Hairs and bristles on thorax black. Five strong dc, four irregular pairs of acr, two strong npl, one strong sa, two strong pa; scutellum with two pairs of sc, medial pair pubescent, lateral pair strong.

Legs black, all coxae and femora black; fore tibia mainly yellow, black dorsally, mid tibia yellow, hind tibia mainly black,yellow dorsally at middle; fore tarsomere 1 inflated apically, fore and mid tarsi black from tip of tarsomere 2 onwards, hind tarsus entirely black. Hairs and bristles on legs black. Fore and mid coxae with bristles, mid coxa apically with a bunch of bristles, hind coxa with one outer bristle. All femora with ventral bristles, mid and hind femora each with one preapical bristle. Fore tibia with five ad, six pd, and four apical bristles, av absent,; mid tibia with two ad, five pd, three av, and four apical bristles; hind tibia with three ad, five pd, three av, and three apical bristles. Relative lengths of tibia and 5 tarsomeres of fore leg 2.5 : 1.2 : 0.7 : 0.4 : 0.3 : 0.4; mid leg 3.2 : 1.7 : 0.8 : 0.6 : 0.5 : 0.4; hind leg 4.3 : 1.3 : 1.2 : 0.8 : 0.5 : 0.4. Wing hyaline, veins black; M bent medially, M and R_4+5_ parallel apically; CuAx ratio 0.5. Squama yellow with yellow hairs. Halter yellow.

Abdomen entirely metallic green with pale gray pruinescence. Hairs and bristles on abdomen black. Male genitalia (Fig. 5): epandrium black, nearly as long as wide. Surstylus black, short and thick, nearly square, with one apical protuberance. Cercus yellow, bifoliate, outer lobe and inner lobe strip-like, inner lobe 1/4 as long as outer lobe, both lobes with short bristles.

Female. Unknown.

#### Types.

Holotype male, CHINA, Qinghai, Gangchaxian, Shaliuhe, 3,200 m; collected by sweep net in grassland, 2015.VIII.06, leg. Liang Wang. Paratypes: one male, same data as holotype.

#### Distribution.

Palaearctic: China (Qinghai).

#### Remarks.

The new species is somewhat similar to *R.heilongjiangense* Wang, Yang & Masunaga, 2005, but the two species can be separated by several features. In *R.gangchanum*, all postocular bristles are black, the thorax has five dc, and the mid femur is black (Fig. 1), the cercus is bifoliate (Fig. 5). In *R.heilongjiangense*, the middle and lower postocular bristles are yellow, the thorax has eight dc, the mid femur is yellow, the cercus is not bifoliate (Yang et al. 2011: 1255, fig. 804).

#### Etymology.

The specific name refers to the type locality, Gangcha.

### Rhaphium shaliuhense sp. n.

Taxon classificationAnimaliaDipteraDolichopodidae

http://zoobank.org/623CD00A-D4C1-44E2-9C60-A21AF345A650

Figures 3 , 6 , 7

#### Diagnosis.

First flagellomere much elongated, about 7.0 times longer than wide; arista basal segment 0.5 times as long as apical segment. All coxae black, all femora mainly black except hind femur yellow ventrally at base. Fore and mid coxae with yellow bristles, mid coxa apically with a bunch of yellow bristles, hind coxa with one yellow outer bristle. CuAx ratio 0.4. Surstylus finger-like, curved apically and rounded, without distinct bristle. Cercus bifoliate, outer lobe strip-like; inner lobe thick, strip-like, apically with three long and strong bristles.

#### Description.

Male (Fig. 3). Body length 2.5 mm. Wing length 2.7 mm.

Head metallic green with pale gray pruinescence. Frons with white pruinescence. Face black with pale pruinescence, not as wide as first flagellomere (length). Upper postocular bristles black, middle and lower postocular bristles yellow. Two oc, two vt, two pvt. Antenna (Fig. 6) black; first flagellomere elongated, about 7.0 times longer than wide, apically sharp; arista black, apical position, basal segment 0.5 times as long as apical segment. Proboscis brown with yellow hairs. Palpus brown with black apical bristle and hairs.

Thorax metallic green with pale gray pruinescence. Mesonotum without dark spot. Hairs and bristles on thorax black. Five strong dc, four irregular pairs of acr, two strong npl, one strong sa, two strong pa; scutellum with two pairs of sc, medial pair pubescent, lateral pair strong.

Legs black; all coxae black, all femora mainly black except hind femur yellow ventrally at base. Hairs and bristles on legs black. Fore and mid coxa with yellow bristles, mid coxa apically with a bunch of yellow bristles, hind coxa with one yellow outer bristle. Mid and hind femora each with one preapical bristle. Fore tibia with one ad, one pd, one av, and two apical bristles; mid tibia with two ad, two pd, one av, and three apical bristles; hind tibia with three ad, four pd, two av, and four apical bristles. Relative lengths of tibia and 5 tarsomeres of fore leg 2.7 : 1.4 : 0.6 : 0.5 : 0.3 : 0.4; mid leg 4.0 : 2.1 : 1.0 : 0.5 : 0.4 : 0.5; hind leg 5.0 : 1.5 : 1.3 : 1.0 : 0.6 : 0.5. Wing hyaline, veins black; M bent medially, M and R_4+5_ parallel apically; CuAx ratio 0.4. Squama yellow with yellow hairs. Halter yellow.

Abdomen entirely metallic green with pale gray pruinescence. Hairs and bristles on abdomen black. Male genitalia (Fig. 7): epandrium black, nearly as long as wide. Surstylus finger-like, curved apically and rounded, without distinct bristle. Cercus bifoliate, outer lobe strip-like, apically with long strong bristles; inner lobe thick, strip-like, apically with three long strong bristles.

Female. Unknown.

#### Types.

Holotype male, CHINA, Qinghai, Gangchaxian, Shaliuhe, 3,200 m, collected by sweep net in grassland, 2015.VIII.06, leg. Liang Wang.

#### Distribution.

Palaearctic: China (Qinghai).

#### Remarks.

The new species is somewhat similar to *R.daqinggouense* Tang, Wang & Yang, 2016, but the two species can be separated by several features. In *R.shaliuhense*, there are four pairs of acr, all coxae are black, and the surstylus is finger-like, curved apically (Fig. 7). In *R.daqinggouense*, there are eight pairs of acr, the all coxae are yellow, except mid coxa is black at basal half, the surstylus is oblanceolate, not curved apically (Tang et al. 2016: 587, fig. 14).

#### Etymology.

The specific name refers to the type locality, Shaliuhe.

### Rhaphium tianshuiense sp. n.

Taxon classificationAnimaliaDipteraDolichopodidae

http://zoobank.org/3F1A436B-AF12-49BA-A19A-7F1A163E7584

Figures 2 , 8 , 9

#### Diagnosis.

First flagellomere about 1.8 times longer than wide; arista with basal segment 0.1 times as long as apical segment. Legs mainly yellow; fore coxa mainly yellow, black basally, mid and hind coxae mainly black, yellow apically; fore femur black dorsally, hind femur black apically; hind tarsus entirely black. Fore coxa with two black bristles, mid coxa with one black bristle at apical half, apically with a bunch of black bristles, hind coxa with one black outer bristle. CuAx ratio 0.52. Surstylus basally thick, apically sharp, with one protuberance. Cercus bifoliate, outer lobe long strip-like, apical curlily with one long strong bristle; inner lobe short, 1/4 as long as outer lobe.

#### Description.

Male (Fig. 2). Body length 3.8–4.0 mm. Wing length 3.1–3.3 mm.

**Figures 8, 9.**
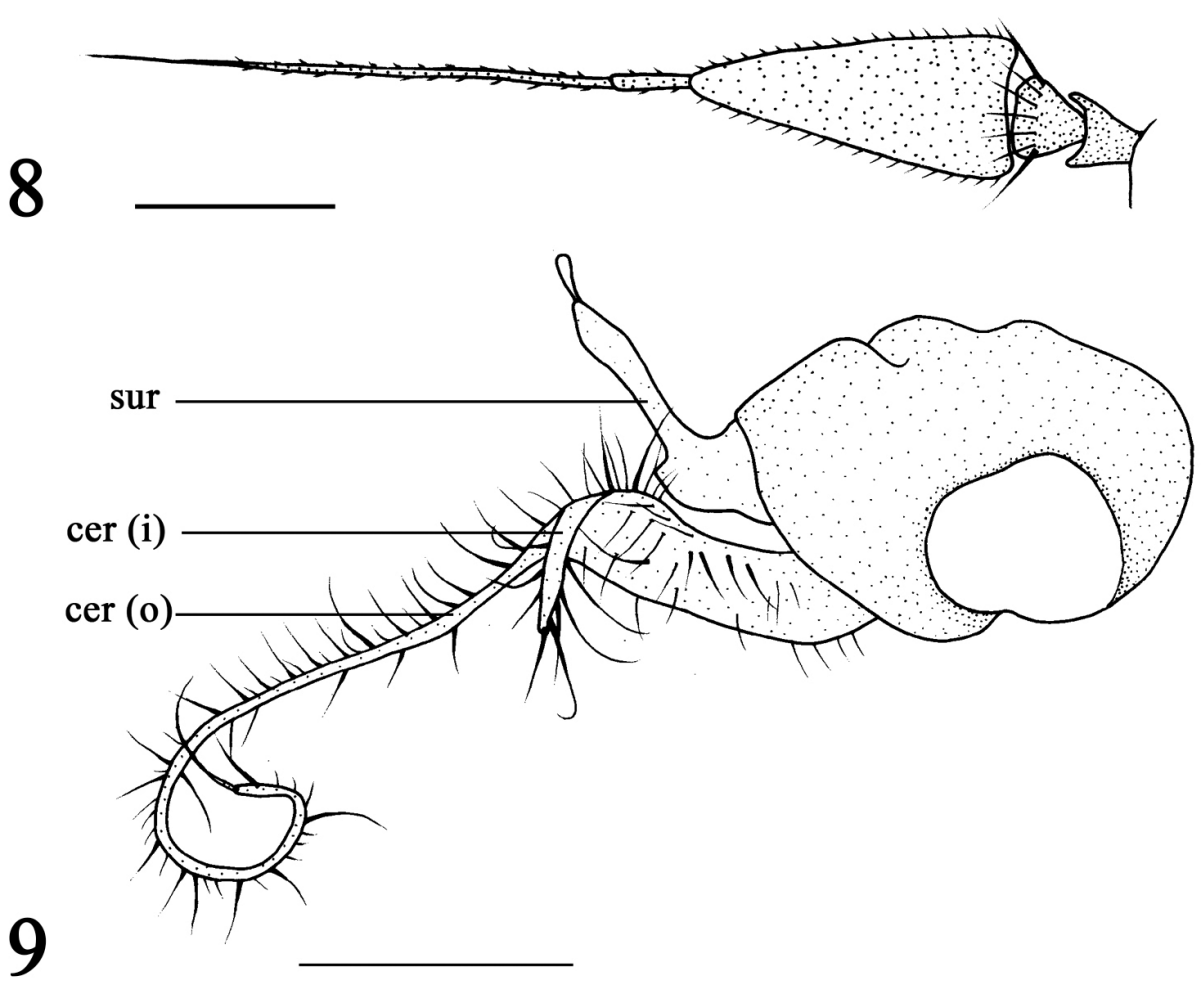
*Rhaphiumtianshuiense* sp. n., male **8** antenna, lateral view **9** Genitalia, lateral view. Abbreviations: sur = surstylus, cer (o) = outer lobe of cercus, cer (i) = inner lobe of cercus. Scale bars: 0.2 mm.

Head metallic green with pale gray pruinescence. Frons with white pruinescence. Face dark metallic green with silvery white pruinescence, not as wide as first flagellomere (length). Upper postocular bristles black, middle and lower postocular bristles yellow. Two oc, two vt, two pvt. Antenna (Fig. 8) black; first flagellomere elongated, about 1.8 times longer than wide, apically sharp; arista black, apical position, basal segment 0.1 times as long as apical segment. Proboscis black with black hairs, palpus black with black apical bristle and hairs.

Thorax metallic green with pale gray pruinescence. Mesonotum without dark spot. Hairs and bristles on thorax black. Five strong dc, four irregular pairs of acr, two strong npl, one strong sa, two strong pa; scutellum with two pairs of sc, medial pair pubescent, lateral pair strong.

Legs mainly yellow; fore coxa mainly yellow, black basally, mid and hind coxae mainly black, yellow apically; fore femur black dorsally, hind femur black apically; fore tarsus black from tip of tarsomere 2 onwards, mid tarsus from tip of tarsomere 3 onward black, hind tarsus entirely black. Hairs and bristles on legs black. Fore coxa with two bristles, mid coxa with one black bristle at apical half, apically with a bunch of black bristles, hind coxa with one black outer bristle. Mid and hind femora each with one black preapical bristle. Fore tibia with two ad, three pd, and two apical bristles, av absent,; mid tibia with three ad, four pd, one av, and three apical bristles; hind tibia with two ad, three pd, and three apical bristles, av absent. Relative lengths of tibia and 5 tarsomeres of fore leg 2.2 : 1.3 : 1.2 : 0.5 : 0.5 : 0.3; mid leg 3.2 : 1.7 : 1.0 : 0.7 : 0.5 : 0.3; hind leg 4.2 : 1.2 : 1.3 : 1.0 : 0.7 : 0.4. Wing hyaline, veins black; M bent medially, M and R_4+5_ parallel apically; CuAx ratio 0.52. Squama yellow with black hairs. Halter yellow.

Abdomen entirely metallic green with pale gray pruinescence. Hairs and bristles on abdomen black. Male genitalia (Fig. 9): epandrium black, nearly as long as wide. Surstylus yellow, basally thick, apically sharp, with one protuberance, without distinct bristles. Cercus bifoliate, outer lobe long strip-like, curled apically with one long strong bristle; inner lobe short, 1/4 as long as outer lobe, apically sharp with strong bristles.

Female. Unknown.

#### Types.

Holotype male, CHINA, Gansu, Tianshui, Maiji Mountain, 1,396 m, collected by sweep net in grassland, 2012.VII.16, leg. Zehui Kang. Paratypes: two males, same data as holotype; four males, Beijing, Miyunxian, Caojialucun, 561 m, collected by sweep net in grassland, 2013.V.23, leg. Xunkun Li.

#### Distribution.

Palaearctic: China (Gansu, Beijing).

#### Remarks.

The new species is similar to *R.mediocre* (Becker, 1922), but the two species can be separated by several features. In *R.tianshuiense*, the squama with black hairs, the surstylus is basally thick, apically sharp, has one protuberance and the cercus is bifoliate (Fig. 9). In *R.mediocre*, the squama with yellow hairs, the surstylus is short and thick, the cercus is not bifoliate (Yang et al. 2011: 1258, fig. 806).

#### Etymology.

The specific name refers to the type locality, Tianshui.

## Discussion

*Rhaphium* is quite a large genus in Dolichopodidae. Negrobov (1986) proposed a key to Palaearctic and Nearctic species of the *R.nasutum* group. Grichanov (2004) and Naglis (2009) mentioned the *R.albifrons* group. Negrobov and Grichanov (2010) published on the *R.crassipes* group. Naglis and Grootaert (2011) published the *R.srilankensis* group. Negrobov et al. (2011) proposed the *R.tridactylum* group. Negrobov et al. (2013) described the *R.ensicorne* group. Tang et al. (2016) mentioned the *R.bilobum* group and *R.flavilabre* group. In having the frons usually with white grey pruinosity, the hind coxa with a strong yellow outer bristle, the fore coxa without a comb of strong bristles, and the cercus usually bifoliated, *R.shaliuhense* sp. n. is included the *R.albifrons* group. The other two new species described in this paper do not match the diagnosis of any known group.

**Figure 10.**
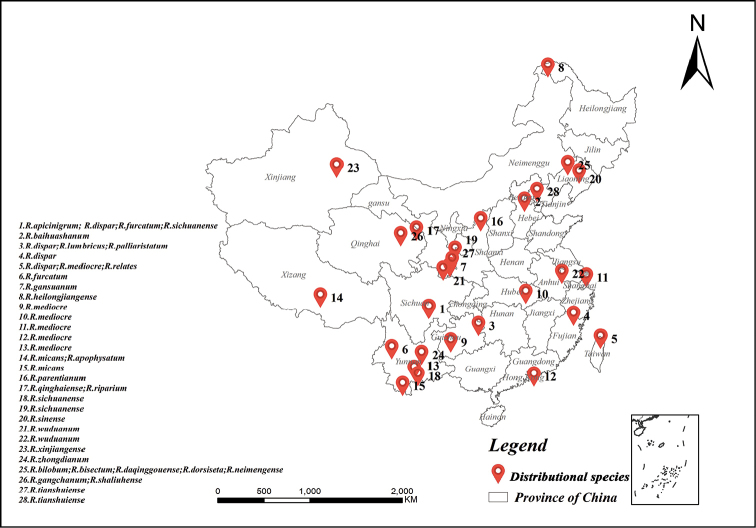
Distribution map with records of all *Rhaphium* species of China (except *R.dilatatum* Wiedemann, 1830 because its distribution is uncertain).

As mentioned, there are 16 species previously recorded from Palaearctic China, and the three new species are all distributed in Palaearctic China. *Rhaphiumgangchanum* sp. n. and *R.shaliuhense* sp. n. are collected from Qinghai province, and *R.tianshuiense* sp. n. is recorded from Gansu Province and Beijing City. *Rhaphium* can be considered a widespread genus in China (Fig. 10). However, Xinjiang, Xizang, and Inner Mongolia have few species, which might be because of the relatively dry climates of these three provinces. Inadequate collection might be another reason.

## Supplementary Material

XML Treatment for Rhaphium

XML Treatment for Rhaphium gangchanum

XML Treatment for Rhaphium shaliuhense

XML Treatment for Rhaphium tianshuiense
